# Control of Drug Dissolution Rate from Film Dosage Forms Containing Valsartan

**DOI:** 10.1155/2016/5135173

**Published:** 2016-05-23

**Authors:** Yoshifumi Murata, Kyoko Kofuji, Chieko Maida

**Affiliations:** Faculty of Pharmaceutical Science, Hokuriku University, Ho-3, Kanagawa-machi, Kanazawa 920-1181, Japan

## Abstract

Film dosage forms (FDs) containing valsartan (VST), a popular antihypertensive drug, were prepared using a casting method with sodium alginate and other polysaccharides as the film base. Drug dissolution profiles of the FDs were investigated in limited medium. The FDs were 170–200 *μ*m thick and were easy to handle. All FDs immediately swelled and disintegrated in the medium. About 23% of the VST incorporated into the FD prepared with 1.5% sodium alginate dissolved at 5 min. The initial dissolution rate of VST increased upon the addition of chitosan to the film base; this effect was not observed in the case of chitin. On the other hand, the rate apparently decreased upon modification with alginic acid. In addition, the solubility of VST in the dissolution medium was changed by the addition of chitosan or alginic acid. FDs prepared with polysaccharides are useful for simplifying the administration of drugs to patients, and the drug dissolution rate from FDs can be controlled by modification.

## 1. Introduction

Film has been noted as an excellent dosage form, especially in oral care. When a film dosage form (FD) is set in a small amount of liquid, it swells quickly and releases the compounds incorporated in the film matrix [1]. FDs can be attached in the oral cavity, allowing drug distribution across the membrane [2, 3]. This can be useful for patients who have difficulty swallowing regular oral dosage forms, such as tablets or capsules [4–6]. However, the utilization of film as a dosage form is limited because the drug loading capacity is low. Furthermore, the drug dissolution profile is typically dependent on the properties of the drug, such as solubility in an aqueous medium. Therefore, control of the drug dissolution rate from the FD is difficult.

It is known that thin films can be prepared using a natural polysaccharide, such as sodium alginate (Alg-Na) or pullulan, without dissolution in organic solvents, heating, or pH regulation. We have already reported the characteristics of FDs prepared with Alg-Na, which has been used widely as a food additive, a tablet disintegrator, or a gelation agent [7]. Moreover, Alg-Na itself is used to treat ulcers by protecting the gastric mucosa.

When preparing the film using the casting method, FD is formed by the evaporation of solvent from the film base solution containing an active compound. FDs prepared with Alg-Na can be modified by additives within the limits of film formation. For example, the drug dissolution rate of miconazole was promoted by the addition of a cyclodextrin to the film base solution [8]. In this study, valsartan (VST), an angiotensin II receptor blocker, was selected as the model drug to be incorporated into an FD [9]. We attempted to modify the FD using alginic acid (ALG) or chitosan (CS) as additives. The drug release profiles from the FDs were also determined in a limited dissolution medium.

## 2. Materials and Methods

### 2.1. Materials

Alg-Na (300 cps) was obtained from Nacalai Tesque Inc. (Kyoto, Japan). Low-molecular-weight Alg-Na (80–120 cps), ALG-S (swelling type), ALG-NS (nonswelling type), and the model drug VST were obtained from Wako Pure Chemicals (Osaka, Japan). Chitosan (CS; degree of deacetylation (DA) 75–85%), CS-F, was obtained from Kimitsu Chemical Industries Co. Ltd. (Tokyo, Japan). CS-K (DA 85%) was obtained from Koyo Chemical Co. (Osaka, Japan) and *β*-CS (DA 96%) from Yaegaki Bio-Industry Inc. (Himeji, Japan). Chitin was obtained from Nacalai Tesque Inc. Pullulan was supplied by Hayashibara Biochemical Laboratories (Okayama, Japan). All other chemicals were of reagent grade.

### 2.2. FD Preparation

Alg-Na (1.5% [w/w]) containing an additive was prepared in deionized water as the film base solution. VST (50 mg) was added with agitation to 10 g of the film base solution. The mixture was thoroughly mixed by sonication, and then 3.0 g of each solution was poured into individual plastic 54 mm Petri dishes. After 24 h at 37°C, the circular films formed on each dish were transferred to a desiccator. Film formation was judged to have failed if a circular film was not obtained, if the film had cracks, or if the film could not be removed from the bottom of the dish. In the present method, 15 mg of VST was theoretically incorporated into each film dosage form.

### 2.3. Film Thickness and Rheological Properties

Thickness was measured at 10 points on each film using a micrometer (CLM1-15QM; Mitutoyo, Kawasaki, Japan) with a set pressure of 0.5 N. Measurements were made using 3 films, and the mean thickness was calculated for each type. The rheological properties of each film were determined using a rheometer (SUN RHEO TEX SD-700#; Sun Scientific Co., Tokyo, Japan) at room temperature. The film was fixed on a vial (inner diameter 1.4 mm, outer diameter 18.8 mm) using a rubber band (Kyowa Co., Osaka) and was probed with a cylindrical adapter (diameter 5.0 mm). Stress and strain were measured at the point at which the adapter broke through the film. The tests were performed in triplicate.

### 2.4. Solubility of VST

The solubility of VST was measured in physiological saline containing ALG-S, CS, or chitin. VST (10 mg) and an additive (10 mg or 20 mg) were added to 20 mL of the test solution and shaken at 37°C for 24 h; then, the suspension was removed using a preheated plastic syringe (Terumo Co., Tokyo) at 37°C and filtered using a syringe driven filter unit (Millex-HV, pore size: 0.45 *μ*m, Millipore Co., MA, USA). The solution was diluted with methanol and injected onto an HPLC column.

### 2.5. Determination of VST

The HPLC system (Hitachi Co., Tokyo) consisted of a pump (L-2130), UV-detector (L-2400), autosampler (L-2200), and chromate-integrator (D-2500) connected to a packed column (150 mm × 4.6 mm, Cosmosil 5C_18_-MS-II, Nacalai Tesque Inc.). To determine the concentration of VST, HPLC was conducted at ambient temperature using an eluent consisting of 10 mM phosphate buffer (pH 3.0), methanol, and acetonitrile (19 : 26 : 5) at a flow rate of 1.0 mL/min [10]. The detector wavelength was set at 230 nm.

### 2.6. VST Dissolution Test

An FD was placed in a plastic dish and 10 mL of the dissolution medium (physiological saline preheated to 37°C) was added. The dish was shaken at 300 rpm in a shaker incubator (SI-300; As One Co., Osaka, Japan) at 37°C. After 1, 3, 5, 10, 15, 20, 30, 45, and 60 minutes, a 0.3 mL aliquot of each solution was removed periodically using a plastic syringe, after which 0.3 mL of the test medium (37°C) was added to maintain a constant volume. The solution was filtered through a syringe driven filter unit (pore size: 0.45 *μ*m). Then, 80 *μ*L aliquots of the filtered solution were placed into micro-test-tubes (1.5 mL) and 720 *μ*L of methanol was added to precipitate the polysaccharide dissolved from the dosage form. Samples were mixed and centrifuged (7,700 ×g, 5 min; H-1300; Kokusan Co., Saitama, Japan), and the supernatants were injected into the HPLC column. All tests were performed in triplicate.

## 3. Results and Discussion

**Figure 1:**
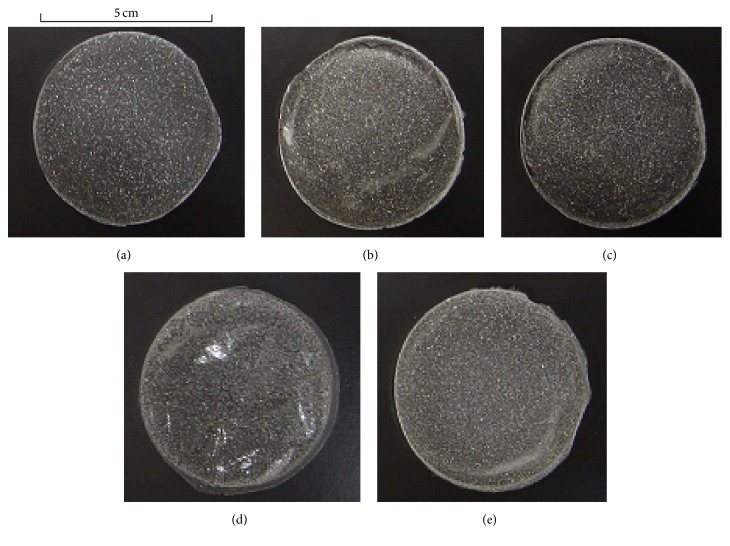
Pictures of FDs prepared with 1.5% Alg-Na and 0.5% additive containing VST: (a) additive-free; (b) ALG-S; (c) ALG-NS; (d) CS-F; (e) chitin.

Using the casting method, an aqueous solution of Alg-Na or pullulan was poured into a Petri dish, and a thin film was formed after evaporation of the solvent. The addition of a drug to the base solution interfered with film formation depending on the polysaccharide. When 0.5% VST was added to a 1.5% Alg-Na solution, a 125 *μ*m thick circular film was obtained with VST homogeneously dispersed. However, pullulan (4–6%) did not form an FD when it contained the same amount of VST. An additive such as ALG-S was added to the film base solution in order to show that the amount of additive also affects FD preparation. As shown in Figure 1, FDs were obtained from the Alg-Na solution containing 0.5% ALG-S or 0.5% ALG-NS, though the films prepared with the solution containing 1% ALG-S, 1% ALG-NS, or 1% CS were cracked. In the case of the 1.5% Alg-Na solution containing each additive, the thickness of the FDs was 170–200 *μ*m, as shown in Table 1.

Because FDs containing VST are used for oral administration, they must be easy to handle as the forms are applied to the oral cavity one by one [11].  Table 2 shows the effect that additives have on the rheological properties of FDs prepared with Alg-Na. All FDs have enough strength to be manipulated by hand. The strength of the FD prepared with 1.5% Alg-Na containing 0.5% chitin could not be measured because of its fragility.

**Figure 2:**
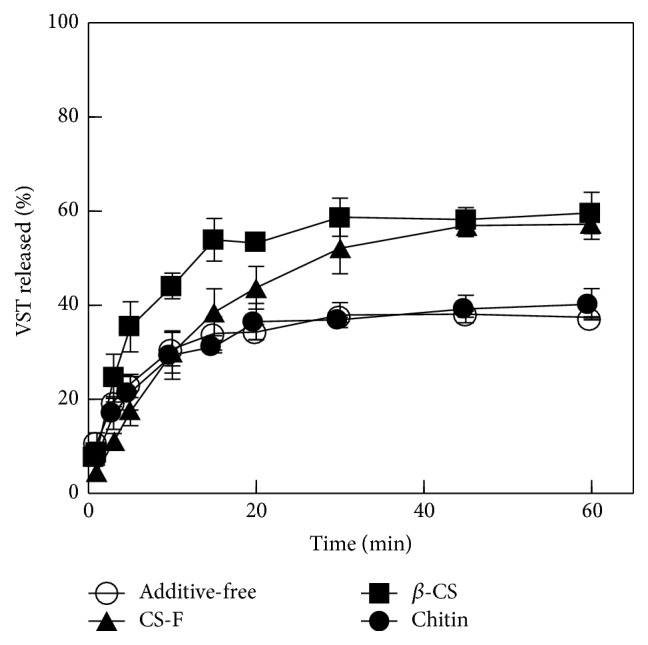
Effect of CS (0.5%) on VST release from FDs prepared with 1.5% Alg-Na in physiological saline.

FDs prepared with a water-soluble polysaccharide immediately swell in physiological saline and then release the drug as they dissolve.  Figure 2 shows the dissolution profiles of VST from FDs prepared with 1.5% Alg-Na. When no additive was used, 3.5 ± 0.2 mg (23 ± 1%) of VST was released from the FD into the test solution at 5 min; the amount was 5.7 ± 0.4 mg (38 ± 3%) at 30 min. When 0.5% CS was added to the base solution, the drug dissolution profile changed. For example, the amount of VST released at 30 min was increased 1.4-fold compared to the control. The initial dissolution rate was especially accelerated by the addition of 0.5%  *β*-CS to film base; the amount of VST dissolved from the FD at 5 min was 5.3 ± 0.8 mg (35 ± 5%). However, the addition of 0.5% chitin to the base solution did not affect the dissolution profile of VST from the FD.

**Figure 3:**
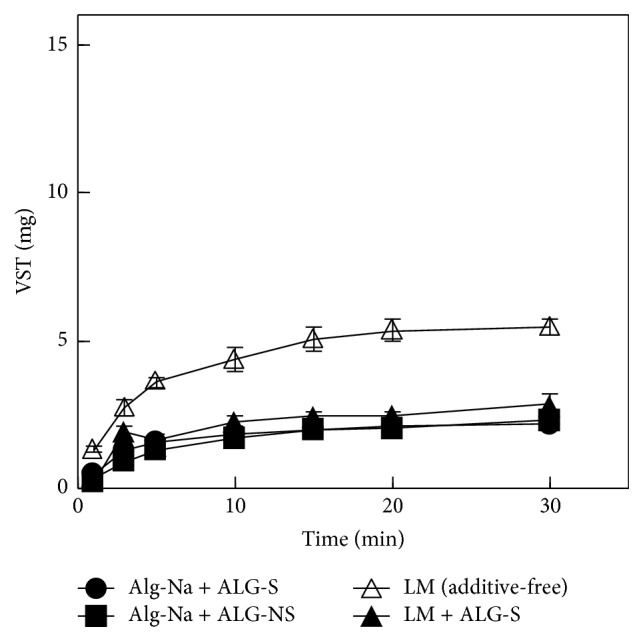
Effect of ALG (0.5%) on VST release from FDs. LM: 2% low-molecular-weight Alg-Na.

On the other hand, the VST dissolution rate from FDs prepared using Alg-Na was decreased by the addition of ALG-S, as shown in Figure 3. The amount dissolved in the test solution at 30 min was 2.2 ± 0.1 mg; this is about 15% of the VST contained in an FD prepared with 1.5% Alg-Na containing 0.5% ALG-S. The drug dissolution rate was also reduced in FDs modified with Alg-NS. Likewise, this phenomenon was observed when FDs were prepared with 2% low-molecular-weight Alg-Na. The VST dissolution from the FD at 5 min decreased from 3.6 ± 0.2 mg to 1.6 ± 0.2 mg with the addition of 0.5% ALG-S to the film base.

Table 3 shows the effect of the additives CS or ALG on VST solubility in physiological saline at 37°C. When a cationic polysaccharide, CS, was added to the test solution, the solubility increased to about 3 times that of the additive-free solution. Conversely, the addition of an anionic polysaccharide, ALG-S, apparently decreased the solubility. In addition, VST solubility remained nearly unchanged by the addition of chitin, an* N*-acetyl derivative of CS. These results show that both CS and ALG affect the drug solubility in physiological saline, though these polymers do not dissolve in the medium. VST has two proton dissociating groups, a carboxyl group and a tetrazole, in the structure, and these parts contribute to the aqueous solubility [12, 13]. Therefore, the change of the drug dissolution rate from the FD may be attributed to an electrostatic interaction between VST and the additive.

**Figure 4:**
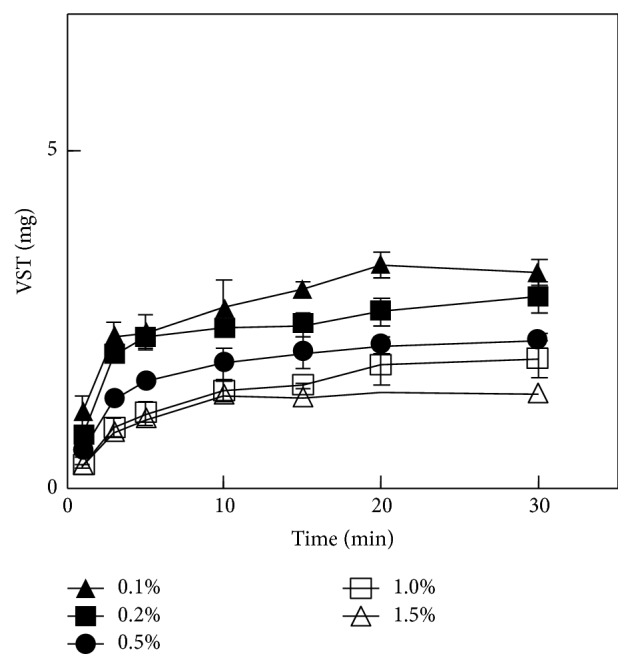
Effect of ALG-S concentration on VST release from FDs prepared with 1.5% Alg-Na.

Figure 4 shows the effects of the additive ALG-S on the VST dissolution rate from FDs. As the amount of ALG-S incorporated into the FD increased, the drug dissolution rate decreased. When 1% ALG-S was added to the base solution, the amount of VST dissolved at 5 min was 1.1 ± 0.2 mg, which is about 7% of the drug incorporated into the FD.

## 4. Conclusions

In this study, FDs were prepared with 1.5–2% Alg-Na as a film base. The FD immediately disintegrated in a limited dissolution medium and released the VST incorporated into the film matrix. The drug dissolution rate from FDs could be controlled by the addition of CS or ALG to the film base solution. VST is a class II drug in the biopharmaceutical classification system because of its low aqueous solubility; therefore, the dissolution profile in the oral cavity may affect the bioavailability [14, 15]. FDs prepared with Alg-Na and additives are useful not only for treating localized problems in the oral cavity, but also for simplifying the administration of drugs to patients.

## Figures and Tables

**Table 1:** Thicknesses of FDs prepared with 1.5% Alg-Na containing 0.5% additive.

Additive	Thickness (*μ*m)
—	125 ± 2
ALG-S	203 ± 14
ALG-NS	198 ± 12
CS-F	180 ± 7
CS-K	174 ± 12
*β*-CS	166 ± 7
Chitin	174 ± 2

**Table 2:** Rheological properties of FDs incorporating VST prepared with 1.5% Alg-Na containing 0.5% additive.

Additive	Stress (kPa)	Strain (mm)
—	151 ± 6	2.3 ± 0.3
ALG-S	206 ± 44	2.9 ± 1.7
ALG-NS	135 ± 49	2.8 ± 0.8
CS-F	145 ± 17	2.8 ± 0.9

**Table 3:** Solubility of VST in physiological saline containing additive at 37°C.

Additive	Solubility (mg/mL)
—	0.16
0.05% CS-F	0.46
0.10% CS-F	0.49
0.05% chitin	0.18
0.10% chitin	0.21
0.05% ALG-S	0.09
0.10% ALG-S	0.05
